# Response to “COVID-19 in persons with haematological cancers”

**DOI:** 10.1038/s41375-020-0914-x

**Published:** 2020-06-11

**Authors:** Stefan Hatzl, Florian Eisner, Gernot Schilcher, Philipp Kreuzer, Maximilian Gornicec, Philipp Eller, Marianne Brodmann, Peter Schlenke, Martin Helmut Stradner, Robert Krause, Hildegard Greinix, Eduard Schulz

**Affiliations:** 1 Division of Hematology, Department of Internal Medicine, Medical University of Graz, Graz, Austria; 2 Intensive Care Unit, Department of Internal Medicine, Medical University of Graz, Graz, Austria; 3 Emergency Medicine Unit, Department of Internal Medicine, Medical University of Graz, Graz, Austria; 4 Division of Angiology, Department of Internal Medicine, Medical University of Graz, Graz, Austria; 5 Department of Blood Group Serology and Transfusion Medicine, Medical University of Graz, Graz, Austria; 6 Division of Rheumatology and Immunology, Department of Internal Medicine, Medical University of Graz, Graz, Austria; 7 Section of Infectious Diseases and Tropical Medicine, Department of Internal Medicine, Medical University of Graz, Graz, Austria

**Keywords:** Haematological cancer, Infectious diseases

We read with interest the article titled “COVID-19 in persons with haematological cancers” by Wenjuan He et al. published recently [1]. The authors conducted a retrospective cohort study of 13 hospitalized patients with hematological malignancies (PHM) from Wuhan, China, who developed coronavirus infectious disease 2019 (COVID-19) and compared their clinical characteristics and outcomes with eleven hospitalized health care providers (HCP). They showed that COVID-19 led to more severe disease and significantly higher case fatality rate (CFR) of 62% in PHM compared to zero in HCP (*P* = 0.002). We acknowledge these findings since data on COVID-19 infections in PHM have been scarce so far, but we see important limitations that were not explicitly addressed in the article. Furthermore, we present our own retrospective cohort data from Graz, Austria, which differs in terms of demographics and outcome.

First, the cohort of PHM with COVID-19 exhibiting a median age of 35 years and selected by lung CT scan is likely not representative for most PHM whose median age reportedly is around 60 years [2, 3]. In Wuhan, PHM with COVID-19 were a median of 14 years younger compared with PHM without COVID-19 (*P* = 0.082). Moreover, 62% (8/13) of PHM with COVID-19 had already been in the intensive care unit (ICU) before the COVID-19 pandemic began. Depending on the reason for ICU admission, which is not specified in the article, mortality rates of up to 60% are to be expected [2]. Taken together, younger but obviously already critically ill patients prior to COVID-19 infection were reported by He et al.

Second, predominantly female HCP with COVID-19 having a median age of 32 years are not an ideal comparator group since in these subjects CFR is expected to be below 2.5% [4]. Only 18% (2/11) of HCP were male compared with 54% (7/13) of PHM with COVID-19 and 57% (65/115) of PHM without COVID-19, respectively (Fisher’s exact test, *P* = 0.0492). It has been previously shown that male gender is strongly associated with worse outcome of COVID-19 [4].

In summary, the expectedly very low CFR in young female HCP and the high mortality in presumably preselected PHM likely overestimated the difference in CFR between these two groups.

We similarly analyzed all consecutive patients infected with SARS-CoV-2 (*N* = 78; Supplementary Table 1) diagnosed at the University Hospital in Graz, Austria, until May 1, 2020. COVID-19 and acute respiratory distress syndrome (ARDS) were diagnosed as previously described [5]. Importantly, all SARS-CoV-2 infections were confirmed by nucleic acid test (NAT) and were not preselected by lung CT scan.

There were eight PHM with a median age of 57 years showing equal sex distribution (Table 1). Surprisingly, there was no difference in overall survival between PHM and subjects without hematologic cancers given the limitation that the latter were 16 years older (Supplementary Fig. 1; Supplementary Table 1). Despite thorough precautions, four PHM (UPN5–8) got infected most likely by asymptomatic HCP demonstrating the danger of nosocomial transmission as has been noted [1]. Two female patients had undergone allogeneic hematopoietic cell transplantation (HCT) 6 months (UPN7) and 5 weeks (UPN8) prior to SARS-CoV-2 infection and had ongoing immunosuppressive therapy including cyclosporine. Overall, six patients developed bilateral pneumonia diagnosed by chest X-ray and of these, three male PHM (UPN3, UPN5–6) progressed to severe ARDS requiring mechanical ventilation in the ICU. Importantly, these three PHM had recently received cytotoxic therapy, two including anti-CD20 antibodies and granulocyte-colony stimulating factors (G-CSF), confirming antitumor treatment <14 days previously as risk factor for severe COVID-19 course [6].

**Table 1:** Demographics, baseline characteristics, and clinical outcomes of hematological patients with SARS-CoV-2 infection.

	UPN1	UPN2	UPN3	UPN4	UPN5	UPN6	UPN7	UPN8
Sex	M	W	M	W	M	M	W	W
Age	61	63	52	64	54	55	58	56
Days from positive NAT to last seen or death	68	66	59	59	23	55	55	55
Days from positive NAT to first symptoms^**a**^	−5	−8	−1	N/A	−2	2	7	10
First symptoms	Fever, cough	Cough, diarrhea	Fever, malaise	None	Fever	Fever	Dyspnea	Dysgeusia, nausea
Hematological disease^**b**^	DLBCL (12 months continuous complete remission)	Coombs positive Evans syndrome, Hodgkin lymphoma (10 year continuous complete remission)	DLBCL	Multiple myeloma	AML-MRC, CRi (severe pancytopenia)	Follicular lymphoma	Lymphoid blast crisis of CML, HLA-identical unrelated donor alloHCT, molecular remission, chronic GvHD	AML-MRC, HLA-identical sibling donor alloHCT, acute skin GvHD
Specific hematologic therapy in the last 12 months before COVID-19 diagnosis	EPOCH-R, high-dose MTX	Eltrombopag^**c**^, prednisolone^**c**^, intravenous immunoglobulins^**c**^	R-CHOP and pegfilgrastim^**c**^	RVD induction; high dose cyclophosphamide (priming therapy) and filgrastim^**c**^	daunorubicin, cytarabine (7 + 3 induction)	G-CHOP and lipegfilgrastim^**c**^	TBI 8 Gy, fludarabine, rabbit ATG, methotrexate (reduced-intensity myeloablative conditioning); dasatinib; methylprednisolone and cyclosporine^**c**^	High-dose cytarabine (consolidation); busulfan, fludarabine (myeloablative conditioning); mycophenolate mofetil and cyclosporine^**c**^
Relevant coexisting disorders	Secondary immunoglobulin deficiency	Iatrogenic Cushing’s syndrome, Parkinson’s disease, severe osteoporosis, recurrent deep vein thrombosis, splenectomy	Obesity	None	Diabetes mellitus, peptic ulcer disease	Chronic obstructive pulmonary disease, arterial hypertension, clear cell renal cell carcinoma (in remission), obesity	Arterial hypertension, QTc-prolongation, extrapulmonary tuberculosis	Arterial hypertension, paroxysmal atrial fibrillation, hyperlipidemia
Smoking history	No	No	No	No	No	Yes, 122 pack years	No	No
All symptoms	Fever, cough, sore throat, respiratory distress	Fever, cough, dyspnea, diarrhea	Fever, mailase, cough, respiratory distress	None	Fever, cough, respiratory distress	Fever, cough, dysgeusia, respiratory distress	Dyspnea, cough, malaise, fever, respiratory distress	Dysgeusia, nausea, cough, fever, respiratory distress
Chest X-ray	Bilateral pneumonia	None	Bilateral pneumonia	None	Bilateral pneumonia	Bilateral pneumonia	Bilateral pneumonia	Bilateral pneumonia
Abnormal blood count^**d**^ before SARS-CoV-2 infection	Lymphopenia	Leukocytosis	Lymphopenia	Severe neutropenia, mild anemia, severe thrombocytopenia, lymphopenia	Severe pancytopenia, lymphopenia	No	Anemia	Anemia, lymphopenia, thrombocytopenia
Laboratory changes since SARS-CoV-2 infection	Increased CRP, d-dimers, ferritin, LDH, PCT (IL-6, sIL-2R not measured)	Lymphocytosis, thrombocytopenia; increased IL-6 (ferritin, sIL-2R not measured)	Neutropenia, thrombocytopenia; increased AST, ALT, CRP, d-dimers, ferritin, HSTT, IL-6, LDH, PCT, sIL-2R	Increased CRP (ferritin, IL-6 and sIL-2r not measured)	Increased CRP, d-dimers, ferritin, HSTT, IL-6, LDH, sIL-2R; decreased fibrinogen	Neutropenia, thrombocytopenia, lymphocytopenia; increased AST, ALT, CRP, d-dimers, ferritin, fibrinogen, HSTT, IL-6, LDH (sIL-2R not measured)	Lymphocytopenia; increased CRP, ferritin, IL-6, sIL-2R	Increased CRP, ferritin, IL-6 (sIL-2R not measured)
Complications	Bacterial pneumonia	None	Severe ARDS, cytokine release syndrome, ventilator associated pneumonia	None	Severe ARDS, extubation failure, cytokine release syndrome, multi organ failure, death	Severe ARDS, cytokine release syndrome	None	CMV reactivation, bacterial enterocolitis
Days to ARDS^**e**^	N/A	N/A	7	N/A	7	6	N/A	N/A
Treatment of COVID-19	Hydroxychloroquine, clarithromycin, zinc	None	Hydroxychloroquine, azithromycin, zinc, tocilizumab (three doses), prednisolone, convalescent plasma with prednisolone	None	Hydroxychloroquine, azithromycin, zinc, tocilizumab (three doses), dexamethasone	Hydroxychloroquine, azithromycin, zinc, tocilizumab (two doses), convalescent plasma with prednisolone	Hydroxychloroquine, zinc	Hydroxychloroquine, azithromycin, zinc
SARS-CoV-2 S1/S2 IgG (EIA) at time of last NAT	N/A	Positive	Weakly positive	N/A	N/A	Negative	Negative	Positive
Days to negative NAT^**f**^	11	N/R	34	7	N/R	48	N/R	N/R
Outcome	Cured, well, discharged	Alive, well, outpatient	Cured, well, rehabilitation	Cured, well, outpatient	Dead	Cured, well, rehabilitation	Alive, well, discharged	Alive, well, discharged

*AlloHCT* allogeneic hematopoietic cell transplantation, *ALT* alanine aminotransferase, *AML-MRC* acute myeloid leukemia with myelodysplasia-related changes, *ARDS* acute respiratory distress syndrome, *AST* aspartate aminotransferase, *ATG* anti-thymocyte globulin, *CRi* complete remission with incomplete hematologic recovery, *CRP* C-reactive protein, *CML* chronic myeloid leukemia, *DLBCL* diffuse large B-cell lymphoma, *EIA* enzyme immunoassay, *EPOCH-R* etoposide, prednisolone, vincristine, cyclophosphamide, doxorubicin, rituximab, *G-CHOP* obinutuzumab, cyclophosphamide, doxorubicin, vincristine, prednisolone, *GvHD* graft versus host disease, *HLA* human leukocyte antigen, *HSTT* highly sensitive troponin t, *IgG* immunoglobulin G, *IL-6* interleukin-6, *LDH* lactate dehydrogenase, *NAT* nucleic acid test, *N/A* not applicable, *N/R* not reached, *PBSC* peripheral blood stem cell, *PCT* procalcitonin, *R-CHOP* rituximab, cyclophosphamide, doxorubicin, vincristine, prednisolone, *RVD* lenalidomide, bortezomib, dexamethasone, *sIL-2R* soluble interleukin-2 receptor, *TBI* total body irradiation, *UPN* unique patient number.

^a^Some patients were identified by NAT screening after contact with infected patient before development of symptoms.

^b^Hematologic disease were classified according to WHO classification.

^c^Administered in the 14 days prior to COVID-19 onset.

^d^Lymphocytopenia was defined as a lymphocyte count of <1000 per cubic millimeter. Thrombocytopenia was defined as a platelet count of <150,000 per cubic millimeter.

^e^Days to ARDS were counted from onset of clinical symptoms.

^f^Days to negative NAT were counted from first positive NAT until the first of two consecutive negative NAT 24 h apart.

Several recurrent laboratory findings have been previously described in patients with COVID-19 [5]. In accordance with prior reports, lymphopenia was observed in seven PHM including five prior to infection. Systemic hyperinflammation was documented in all but one PHM (UPN4), but compared to patients without hematologic cancers, PHM showed significantly higher peak levels of IL-6 (median of 1207 vs. 36.4 pg/mL, *P* = 0.033) and serum ferritin (3756 vs. 558 ng/mL, *P* < 0.001) but not CRP (Fig. 1a). The latter is in contrast to He et al. reporting significantly higher CRP and procalcitonin levels possibly associated with high bacterial and fungal coinfection rates that resulted in the high CFR [1].

**Fig. 1:**
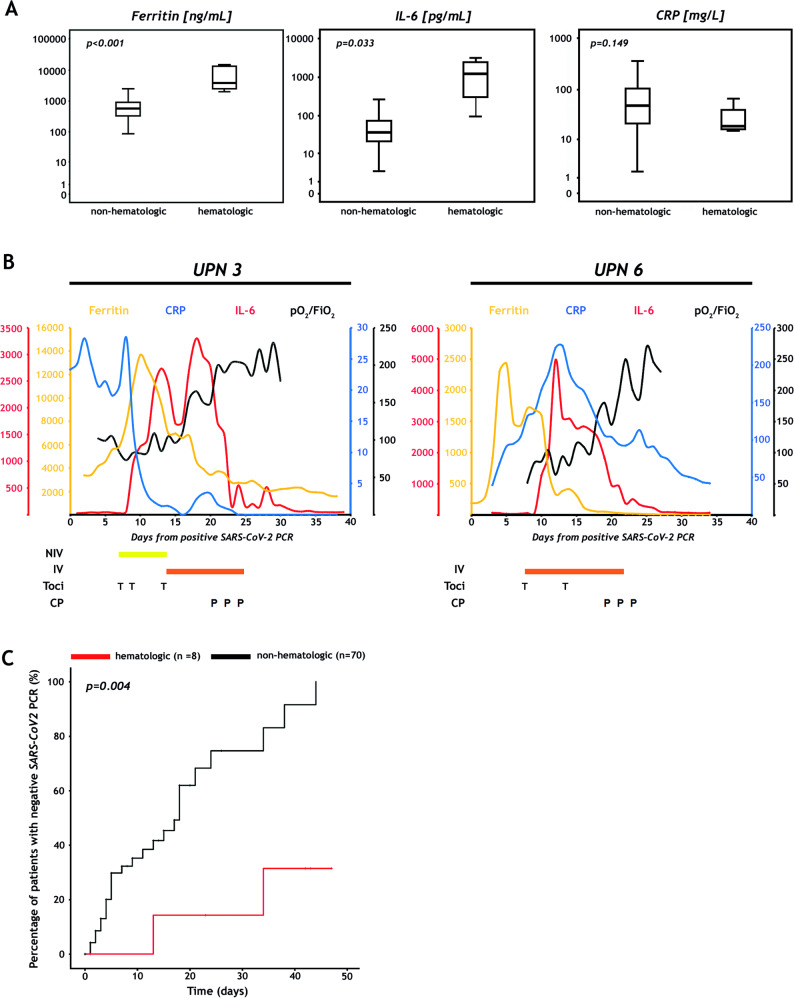
Inflammatory markers, clinical courses after SARS-CoV-2 convalescent plasma administration and time to negative nucleic acid test in patients with hematologic malignancies and COVID-19. **a** Comparison of inflammatory markers in patients with and without hematologic malignancies. Box plots display serum ferritin (left), interleukin-6 (IL-6; middle), and C-reactive protein (CRP; right) in eight hematologic versus 70 non-hematologic patients on log-transformed *y*-axis. The maximum values were used in every subject, except in patients who received tocilizumab. Here, the maximum values before tocilizumab infusion were selected because serum ferritin and IL-6 would regularly increase, and CRP would decrease after tocilizumab administration. *P*-values were calculated with the Kruskal–Wallis test (see Supplementary information). **b** Clinical courses of patients UPN3 and UPN6 receiving SARS-CoV-2 convalescent plasma. Serum ferritin (ng/mL), CRP (mg/L), IL-6 (pg/mL) and the ratio of partial pressure of oxygen in blood (PaO_2_ in millimeters of mercury) and the fraction of oxygen in the inhaled air (FiO_2_) are depicted as a measure of inflammation and respiratory function, respectively. Tocilizumab was administered at a dose of 8 mg/kg body weight. Patients received 200 mL of ABO compatible SARS-CoV-2 convalescent plasma (CP) every other day for three times. CP was collected by standard apheresis and further pathogen-inactivated by INTERCEPT Blood System (Cerus, B.V. Europe). IV invasive mechanical ventilation, NIV non-invasive ventilation, P convalescent plasma, T tocilizumab, UPN unique patient number. **c** Time to SARS-CoV-2 qRT-PCR negativity in hematologic and non-hematologic patients. Analysis was performed with competing risk cumulative incidence estimators and Gray’s tests (see Supplementary information). Data cut off was May 12, 2020. The black line indicates non-hematologic patients, the red line patients with hematologic diseases.

The experimental pharmacological therapy of COVID-19 and treatment outcomes are summarized in Table 1. The two PHM after HCT had a surprisingly unremarkable clinical course. Patients with ARDS also developed cytokine release syndrome and were treated with tocilizumab but no significant improvement of the respiratory situation could be achieved [7]. Therefore, we decided to administer SARS2-CoV-2 convalescent plasma (CP) on a compassionate use basis to patients UPN3 and UPN6, 6 and 11 days after start of mechanical ventilation, respectively [8]. As shown in Fig. 1b, in both cases, IL-6 and serum ferritin decreased dramatically, and patients were off the ventilator 5 and 4 days after CP therapy, respectively. UPN3 and UPN6 achieved a negative NAT whereas another three PHM remained positive after a median time of 55 days. Not surprisingly, compared with subjects without hematologic cancers, PHM needed significantly longer to achieve negative NAT (Table 1; Fig. 1c). Production of anti-SARS-CoV-2 antibodies, which reportedly occur in all COVID-19 patients 19 days after symptom onset, did not always accompany negative NAT in PHM [9]. After a median follow-up of 57 days, one patient (UPN5) died of severe ARDS, two patients (UPN3, UPN6) are in rehabilitation centers without symptoms of COVID-19, and five have been discharged.

Previous studies displayed cancer patients as vulnerable population with high risk of morbidity due to COVID-19 [1, 6, 10]. In our observation, CFR of COVID-19 in PHM is lower than reported by He and co-workers (13% vs. 62%). Nevertheless, we must consider patient heterogeneity and admit that empirical knowledge about the experimental treatment of COVID-19 has evolved since the first outbreak in Wuhan, China. Whether additional administration of anti-CD20 therapy and G-CSF had impact on hyperinflammation and development of ARDS must be assessed in studies with larger patient numbers. As shown in two PHM with severe ARDS due to COVID-19, treatment with CP seems to be promising but requires further evaluation in randomized controlled trials.

## Supplementary information

Supplementary information
