# Supplementary material for: Response to “COVID-19 in persons with haematological cancers”
Source: Leukemia. 2020 Jun 11;34(8):2265–70. doi: 10.1038/s41375-020-0914-x (PMC7289538; doi:10.1038/s41375-020-0914-x)
Supplement: Supplementary file 1 — Supplementary information [file 41375_2020_914_MOESM1_ESM.pdf]

Supplementary information

Response to “COVID-19 in persons with haematological cancers”

Stefan Hatzl et al.

- Statistical methods
- Supplementary Table 1. Baseline characteristics of the study population (N=78).
- Supplementary Figure 1. Overall survival of the study population - Kaplan-Meier curves displaying hematologic versus non-hematologic patients (N=78).

Statistical methods

Patient data cut off for statistical analyses was May 12, 2020. All statistical analyses were performed using SPSS 23.0 (SPSS Inc., Chicago, IL, USA) and R 3.6.1 (https://www.r-project.org/), respectively. All statistical tests were performed two-sided, and a P-value of <0.05 was considered statistically significant.

Kruskal-Wallis test was used for comparison of serum ferritin, interleukin-6 as well as C-reactive protein between hematologic and non-hematologic patients. Count data and continuous variables of baseline characteristic were compared using ranksum-tests, Fisher's exact tests, and $\chi 2$-tests, as appropriate.

Co-primary outcomes included risk of SARS-CoV-2 qRT-PCR (quantitative reverse transcription polymerase chain reaction) negativity and overall survival. Overall survival was analyzed with Kaplan-Meier estimators and log-rank tests whereas risk of SARS-CoV-2 qRT-PCR negativity was analyzed with competing risk cumulative incidence estimators, Gray's tests (1).

Reference:

(1) Dignam JJ, Kocherginsky MN. Choice and interpretation of statistical tests used when competing risks are present. J Clin Oncol. 2008;26(24):4027-4034. doi:10.1200/JCO.2007.12.9866

Supplementary Table 1

Variable	N (%missing)	Overall (N=78)	Hematologic (N=8)	Non-hematologic (N=70)	P-value
**Demographic**					
Age (years)	78 (0%)	69 [55-80]	57 [54-60]	73 [57-80]	**0.035**
BMI (kg/m²)	62 (80%)	25.0 [24.3-27.7]	26.0 [24.3-28.1]	24.7 [23.9-27.6]	0.597
Male	78 (0%)	37 (47%)	4 (50%)	33 (47%)	0.584
**Co-morbidities**					
Hypertension	78 (0%)	36 (46%)	3 (38%)	33 (47%)	0.719
Diabetes	78 (0%)	13 (16%)	1 (13%)	12 (17%)	0.601
Atrial fibrillation	78 (0%)	15 (19%)	1 (13%)	13 (18%)	0.427
Coronary heart disease	78 (0%)	14 (13%)	1 (13%)	13 (19%)	0.560
Cardiomyopathy of any cause	78 (0%)	8 (10%)	0 (0%)	8 (11%)	0.403
Vascular disease	78 (0%)	8 (10%)	1 (13%)	7 (10%)	0.597
Prior thromboembolic event	78 (0%)	5 (7%)	1 (13%)	4 (6%)	0.425
Chronic hemodialysis	78 (0%)	5 (7%)	0 (0%)	5 (7%)	0.573
COPD	78 (0%)	6 (8%)	1 (13%)	5 (7%)	0.490
Lung disease of other cause	78 (0%)	4 (5%)	0 (0%)	4 (6%)	0.643
Prior solid cancer	78 (0%)	5 (7%)	1 (13%)	4 (6%)	0.427
Active solid cancer	78 (0%)	6 (8%)	0 (0%)	6 (9%)	0.520
Dementia	78 (0%)	10 (13%)	0 (0%)	10 (14%)	0.310
**Outcomes**					
ARDS (any grade)	78 (0%)	33 (42%)	3 (38%)	30 (43%)	0.310
ICU admission	78 (0%)	21 (27%)	3 (38%)	18 (26%)	0.368
Duration of ICU stay (days)	21 (0%)	17 [4.0-18.0]	20 [19.0-22.0]	10 [4.0-17.8]	0.080
Time to ICU admission (days)	21 (0%)	3 [0.0-7.0]	6 [5.5-7.0]	2 [0.0-7.0]	0.262
Deceased at data cut of May 12, 2020	78 (0%)	19 (24%)	1 (13%)	18 (26%)	0.371

Supplementary Table 1. Baseline characteristics of the study population (n=78). Data are reported as medians [25th-75th percentile] for continuous variables and absolute frequencies (%) for count data. N (% miss.) reports the number of patients with fully observed data (% missing). Abbreviations: BMI – Body Mass Index, COPD – chronic obstructive lung disease, ARDS – acute respiratory distress syndrome, ICU – intensive care unit.

Supplementary Figure 1

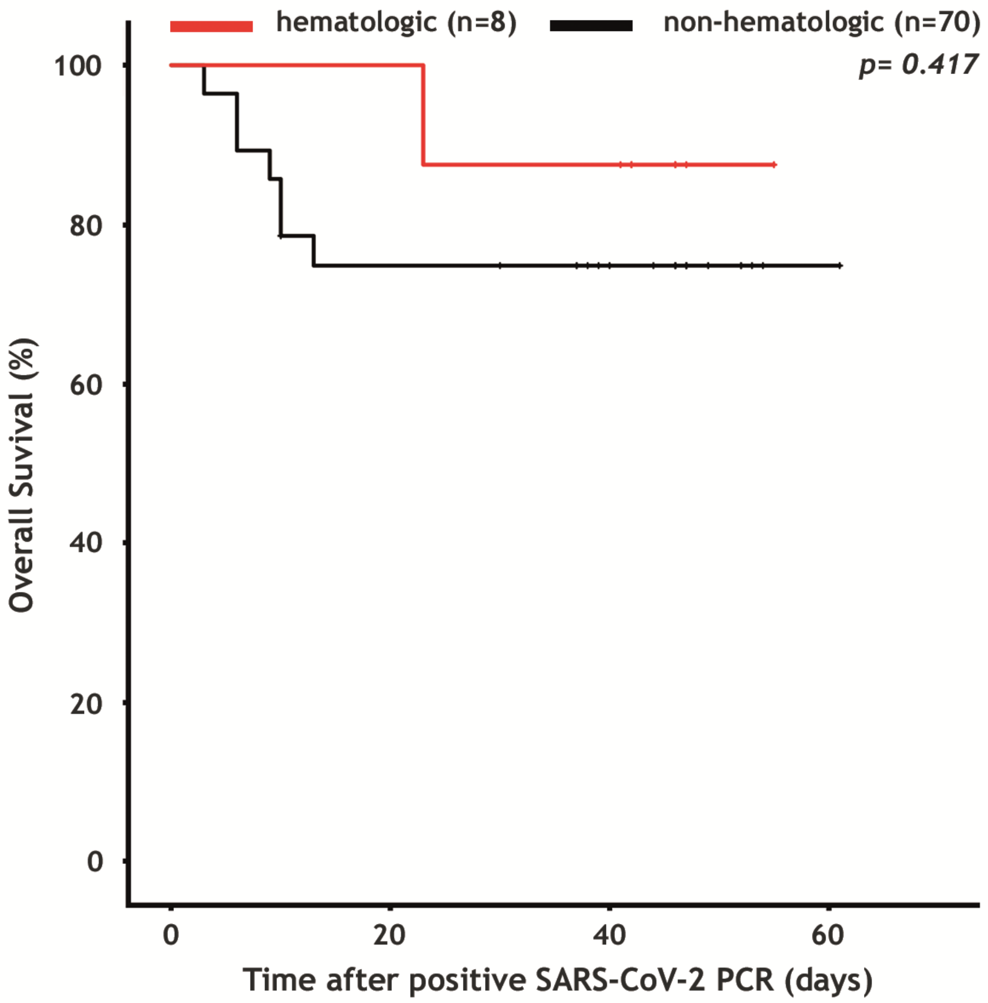

Supplementary Fig. 1 Overall survival of the study population - Kaplan-Meier curves displaying hematologic versus non-hematologic patients (N=78). Red line - hematologic patients, black line - non-hematologic patients.
